# The Decision of Judge Townsend

**Published:** 1900-04

**Authors:** 


### Editorial.

### THE DECISION OF JUDGE TOWNSEND.

There will be found upon another page of this number the decision of Judge Townsend in the case of the International Tooth-Crown Company *vs.* James Orr Kyle. As far as our reading goes this has not been given in full in any of the journals. There may be good reasons for this not appearing upon the surface, but as far as this journal is concerned a copy could not be procured until the present time.

In this connection and intimately associated with it, the reply of the Newark, N. J., dentists, who accepted the Crown Company's conditions, is also given. It is an answer to the criticisms of the *Dental Digest* by one of those interested, but it is presumed that it outlines the sentiments of all. It is given publicity in justice to the parties interested, the editor reserving the right to agree or disagree with the course which the writer has seen fit to adopt.

The decision of the judge includes some statements that seem to require explanation from the chairman of the Dental Protective Association.

In quoting from Judge Wheeler in regard to the testimony of Dr. Beardslee, Judge Townsend says, "In the present suit not one of these witnesses is able positively to identify said exhibit, and the 'wife of a clergyman and her two daughters' now testify, after an examination of church records, etc., that they were mistaken in their former testimony, and that the cap was not put into Mrs. Martz's mouth until 1878, or until after the Low invention was completed, as found in the Richmond case and further proved herein. Even Dr. Beardslee now says that he cannot now testify that said work was done any earlier than the year 1878, and that, so far as he knows, the testimony of the Martzes as to the date when it was done is correct. And further, as if to cap the climax of these contradictions, an apparently disinterested witness, Dr. Palmer, testified that he himself made the Beardslee-Martz exhibit, and was told at the time that, 'whatever of the kind I did was for use in defending the suit of the International Crown Company.' It is unnecessary to further discuss this branch of the case."

Now, what can be the meaning of this? Are we to infer that these witnesses are the ones upon whom the Dental Protective Association relied in previous suits with the Crown Company, and that for some cause, not understood, have suddenly become afflicted with defective memories? No doubt the chairman of the Dental Protective Association can explain this; at all events, many would be pleased to know whether previous decisions were based upon this apparently unreliable testimony. It is thought that, in justice to the subscribers to the protective fund, a certain degree of frankness in dealing with this subject is their due. It is well understood, however, that the position of the executive officer of the Protective Association is a delicate one, and necessarily requires a wise withholding of matters that might prove of too great interest to the Crown Company.

The members of the Protective Association have had entire confidence in the chairman of that organization, and this has not been changed by Judge Townsend's decision. They have not been willing to accept the damaging charges freely circulated by the enemies of the association as worthy of credence.

The fact that the Crown Company does not seem to be making much progress in their crusade leads to the supposition that they prefer not to take the risk of another suit.

The concluding portion of the judge's decision will, perhaps, throw some light upon this; at all events, it may lead the Crown Company to hesitate before making a second contest in which relationship does not exist.

The judge stated that, "At the conclusion of the first day's argument, counsel for the complainant for the first time learned that the defendant herein was related to one of the officers of the complainant's corporation, and that one of its stockholders had contributed to the defence herein without the knowledge of counsel for defendant. Counsel for complainant at once fully and frankly brought this matter to the attention of the court and asked to be advised thereon."

